# Baseline quasispecies selection and novel mutations contribute to emerging resistance-associated substitutions in hepatitis C virus after direct-acting antiviral treatment

**DOI:** 10.1038/srep41660

**Published:** 2017-01-30

**Authors:** Yugo Kai, Hayato Hikita, Naoki Morishita, Kazuhiro Murai, Tasuku Nakabori, Sadaharu Iio, Hideki Hagiwara, Yasuharu Imai, Shinji Tamura, Syusaku Tsutsui, Masafumi Naito, Meiko Nishiuchi, Yasuteru Kondo, Takanobu Kato, Hiroshi Suemizu, Ryoko Yamada, Tsugiko Oze, Takayuki Yakushijin, Naoki Hiramatsu, Ryotaro Sakamori, Tomohide Tatsumi, Tetsuo Takehara

**Affiliations:** 1 Department of Gastroenterology and Hepatology, Osaka University Graduate School of Medicine, Suita, Japan; 2 Hyogo Prefectural Nishinomiya Hospital, Nishinomiya, Japan; 3 Kansai Rosai Hospital, Amagasaki, Japan; 4 Ikeda Municipal Hospital, Ikeda, Japan; 5 Minoh City Hospital, Minoh, Japan; 6 Itami City Hospital, Itami, Japan; 7 Suita Municipal Hospital, Suita, Japan; 8 Saso Hospital, Nishinomiya, Japan; 9 Department of Hepatology, Sendai Kousei Hospital, Sendai, Japan; 10 Department of Virology II, National Institute of Infectious Diseases, Tokyo, Japan; 11 Department of Laboratory Animal Research, Central Institute for Experimental Animals, Kawasaki, Japan; 12 Osaka Rosai Hospital, Sakai, Japan

## Abstract

Resistance-associated substitutions (RASs) in hepatitis C virus (HCV) appear upon failure of treatment with direct-acting antivirals (DAAs). However, their origin has not been clarified in detail. Among 11 HCV genotype 1b patients who experienced virologic failure with asunaprevir (ASV)/daclatasvir (DCV), 10 had major NS5A L31M/V-Y93H variants after treatment. L31M/V-Y93H variants were detected as a minor clone before therapy in 6 patients and were the most closely related to the post-treatment variants by phylogenetic tree analysis in 4 patients. Next, to consider the involvement of a trace amount of pre-existing variants below the detection limit, we analysed human hepatocyte chimeric mice infected with DAA-naïve patient serum. L31V-Y93H variants emerged after treatment with ledipasvir (LDV)/GS-558093 (nucleotide NS5B inhibitor) and decreased under the detection limit, but these variants were dissimilar to the L31V-Y93H variants reappearing after ASV/DCV re-treatment. Finally, to develop an infection derived from a single HCV clone, we intrahepatically injected full-genome HCV RNA (engineered based on the wild-type genotype 1b sequence) into chimeric mice. A new Y93H mutation actually occurred in this model after LDV monotherapy failure. In conclusion, post-treatment RASs appear by 2 mechanisms: the selection of pre-existing substitutions among quasispecies and the generation of novel mutations during therapy.

The treatment of chronic hepatitis C has recently and greatly changed due to the introduction of direct-acting antivirals (DAAs), which remarkably improve the sustained virologic response (SVR)^1^. However, resistance-associated substitutions (RASs) emerge in patients with virologic failure (VF) after IFN-free DAA treatment to create new complications. DAA combination therapy, which includes first generation non-structural (NS) 5A inhibitors, such as daclatasvir (DCV)^2^,^3^, ledipasvir (LDV)^4^,^5^,^6^,^7^ and ombitasvir^8^,^9^,^10^, represents a common regimen for HCV treatment. However, RASs, especially L31M/V and/or Y93H in the NS5A region, are frequently observed after VF because of their low genetic barrier^2^,^3^,^9^,^10^,^11^. An *in vitro* investigation using a replicon system revealed that linked L31M/V-Y93H double substitutions in the NS5A region have extremely high resistance against NS5A inhibitors such as DCV or LDV^12^,^13^,^14^,^15^. Therefore, the emergence of a L31M/V-Y93H double substitution during DAA treatment (which includes a NS5A inhibitor) could play a prominent role in VF. However, no detailed analyses have been performed to determine whether both the L31M/V and Y93H substitutions were present in a single HCV clone in hepatitis C patients.

The mechanisms for emerging HCV RASs through DAA treatment failure in chronic hepatitis C patients have also not been addressed in detail. Quasispecies were reported in hepatitis C patients; thus, diversity and heterogeneity were present in the viral genome within each hepatitis C patient’s serum samples^16^. Additionally, RNA viruses, such as HCV, have extremely high mutation rates^17^. In light of the HCV viral quasispecies and high viral mutation rates, two possible mechanisms arise for emerging RASs during DAAs treatment. These mechanisms include the selection of pre-existing substituted variants in quasispecies and new additional mutations during DAA treatment. To assess these mechanisms, it is necessary to investigate the RASs in quasispecies at baseline and evaluate their changes during DAA treatment failure.

In this study, we examined NS5A L31M/V-Y93H double substitutions in one amplicon using deep sequencing without fragmentation and then used phylogenetic tree analysis to estimate the origin of L31M/V-Y93H double substitutions that emerged after DAA treatment. In the first portion, we revealed for the first time that L31M/V-Y93H double substitutions in patients with VF after ASV/DCV treatment were derived from pre-existing L31M/V-Y93H double substituted variants, which occurred in nearly half of the cases. However, these variants were newly generated in the remaining cases. In the following section, we demonstrated that the extremely rare L31V-Y93H double substituted variants under the detection limit did not contribute to L31V-Y93H double substitutions after DAA re-treatment using human hepatocyte chimeric TK-NOG mice. In the final section, we established a monoclonal wild HCV-infected mouse model and confirmed that the NS5A Y93H mutation was newly generated without quasispecies by the NS5A inhibitor LDV treatment in an *in vivo* situation. Through these experiments, we elucidated that both mechanisms, the selection from quasispecies and the generation of new mutations, contribute to emerging L31M/V-Y93H double substitutions after DAA treatment.

## Results

### NS5A L31 and/or Y93 substitutions before and after ASV/DCV treatment

Among 322 hepatitis C patients who received ASV/DCV treatment, 14 with genotype 1b who had never experienced DAA treatment developed VF. Of these 14 patients, 3 did not succeed in generating PCR amplicons and 11 were analysed using deep sequencing at both baseline and after VF (Table 1). The amino acid at NS5A L31 and Y93 were analysed sequentially in each amplicon, and the frequency of L31-Y93 wild type, L31M/V-Y93 single substitution, L31-Y93H single substitution and L31M/V-Y93H double substitution were calculated. After VF, one patient (case 11) had neither L31 nor Y93 substitutions and had an NS5A P32 deletion at 99.7%, while the other 10 patients had L31M/V-Y93H double substitutions at high frequencies. The patient who lacked the L31-Y93 substitution (case 11) was excluded from the analysis investigating the origin of the L31M/V-Y93H double substitution after VF. Among the remaining 10 patients, 8 had at least one of either a L31M/V or Y93H single substitution or L31M/V-Y93H double substitution at various frequencies at baseline; cases 9 and 10 did not. Interestingly, 6 of the 10 patients had a L31M/V-Y93H double substitution at baseline. Among these 6 patients, 2 had a L31M/V-Y93H double substitution at baseline as a minor clone over 10% and 4 patients had it as a minor clone at less than 1% (Table 1).

### The origin of a L31M/V-Y93H double substitution in patients after VF via ASV/DCV

**Figure 1:**
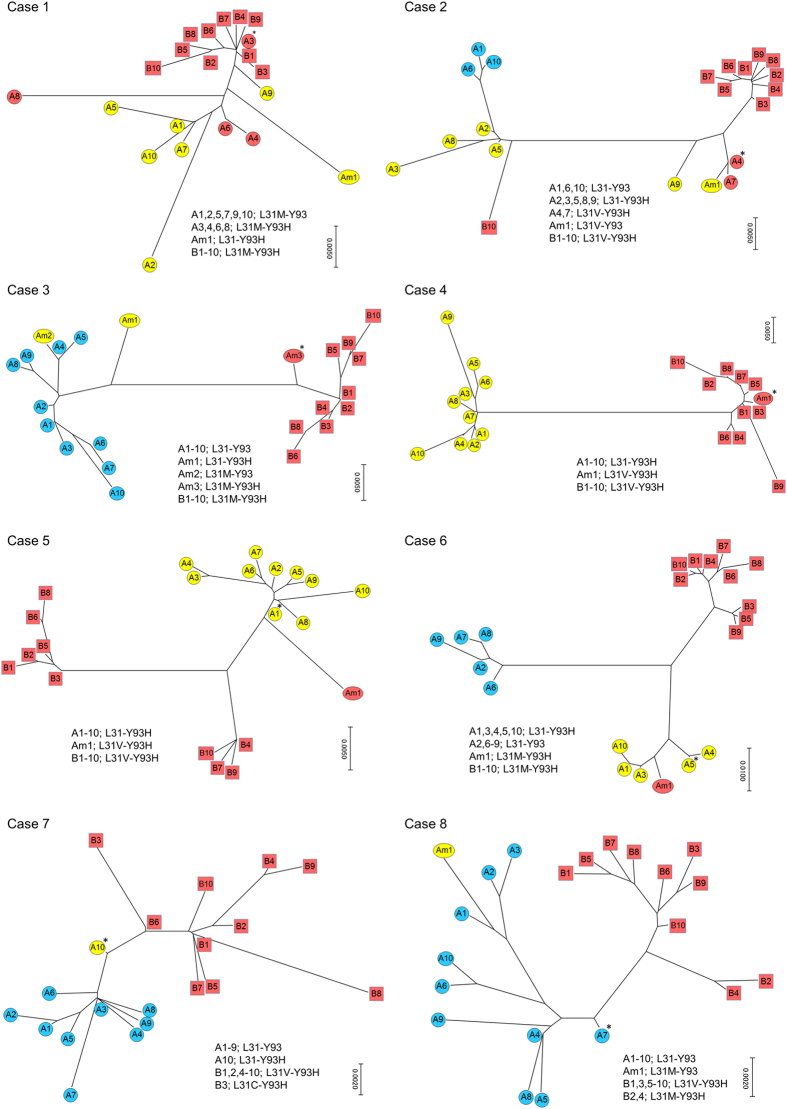
Phylogenetic tree analysis of the non-SVR patients with ASV/DCV. In each case, the top 10 clusters from the HCV clones at baseline are represented as A1 to A10 with coloured circles, and the top 10 clusters at VF are represented as B1 to B10 with coloured squares. Blue, yellow and red colours represent the L31-Y93 wild type, L31M/V or Y93H single substitution and L31M/V-Y93H double substitution, respectively. The top minor substituted cluster at baseline was also represented with an Am in the phylogenetic tree (for example, Am1). *Represents the putative original cluster before treatment that contributed to the L31M/V-Y93H double substitution after treatment.

We performed phylogenetic tree analysis on cases 1 to 8 to investigate the origin of a double substitution after VF. These cases displayed a mixture of different HCV patterns in their L31-Y93 substitutions (Fig. 1). In case 1, the phylogenetic tree was constructed using the top 10 clusters from the baseline and post-relapse (A1–10 and B1–10, respectively) together with the top 1 cluster from the L31-Y93H (Am1) minor clone. The constructed tree revealed that A3, which was the cluster that included the L31M-Y93H double substituted variants at baseline, was closely related to B1 to B10, which were the clusters that included the post-treatment L31M-Y93H double substituted variants. This result suggested that the L31M-Y93H double substituted variants at baseline were selected and increased during ASV/DCV therapy. In other words, the pre-existing L31M-Y93H double substituted variants were the putative origin for the double substitution after VF. Similarly, in cases 2, 3 and 4, the phylogenetic trees revealed that the clusters for the L31M/V-Y93H double substituted variants at baseline (A4, Am3, and Am1, respectively) were closely related to the post-treatment double substituted clusters, suggesting that the pre-existing double substituted variants were the putative origin of the double substitutions after VF, which was similar to case 1. In contrast, in cases 5, 6 and 7, the phylogenetic trees revealed that the baseline L31-Y93H single substituted clusters (A1, A5 and A10, respectively) were closely related to the post-treatment double substituted clusters, suggesting that the baseline L31-Y93H single substituted variants were added with a new mutation of L31M/V, which led to the double substitutions after VF. In case 5 and 6, the minor L31M/V-Y93H double substituted clusters were existed at baseline but not the putative origin of the double substitutions after VF. As an additional investigation, not only the top 1 minor cluster but also all quite minor clusters which consisted of more than 3 leads from pre-existing L31M/V-Y93H double substituted variants were re-examined using phylogenetic trees (Supplementary Figure 1). The constructed trees, including quite minor clusters, also confirmed that these quite minor pre-existing L31M/V-Y93H clusters were not the putative origin of the double substitutions after VF in case 5 and 6. In case 8, the phylogenetic tree revealed that the baseline L31-Y93 wild cluster for A10 was closely related to the post-treatment double substituted clusters, suggesting that the baseline wild clones were added with new mutations of both L31M/V and Y93H, which generated double substitutions after VF.

### The influence of rare substituted variants below the detection limit using HCV-infected mice

**Figure 2:**
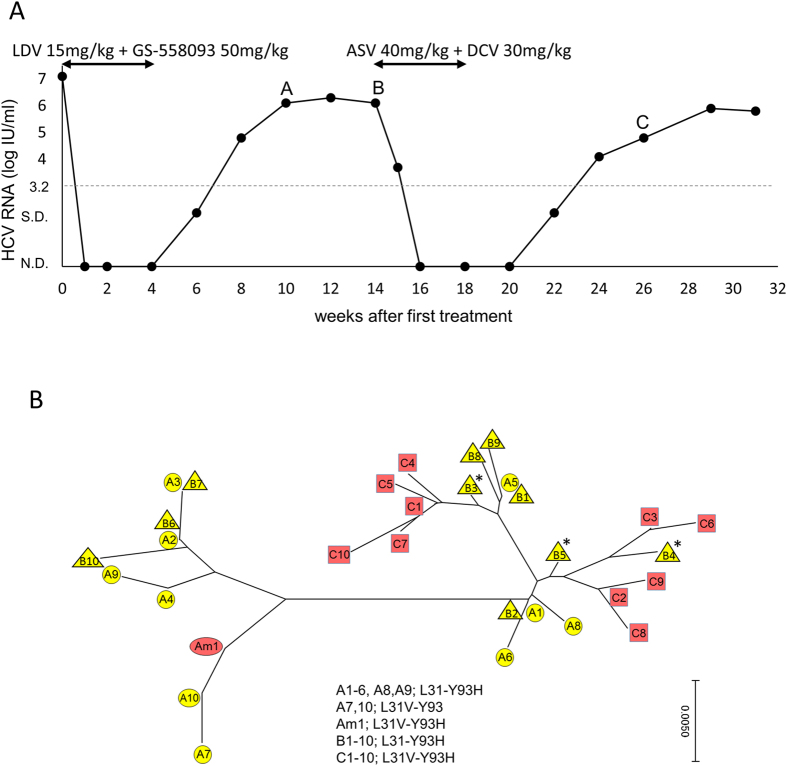
Phylogenetic tree analysis of the non-SVR mouse with ASV/DCV. Human hepatocyte chimeric mice that experienced relapse after LDV/GS-558093 treatment and re-treatment with ASV/DCV. Serum HCV RNA levels (**A**). N.D., not detected, and S.D., signal detected but at lower quantification levels. Deep sequencing was performed at weeks 10 (point A), 14 (point B) and 26 (point C). The phylogenetic tree was constructed with the top 10 clusters at each time point (A1 to A10 with circles, B1 to B10 with triangles and C1 to C10 with squares) and the top cluster from the minor L31V-Y93H double substitution at week 10 (Am1) (**B**). *Represents the putative original cluster before ASV/DCV re-treatment that contributed to the L31V-Y93H double substitution after re-treatment.

To evaluate the influence of the extremely rare substituted variants that are below the detection limit of deep sequencing on emerging RASs during DAA treatment, we used HCV-infected human hepatocyte chimeric TK-NOG mice. We previously reported that the serum HCV RNA levels of chimeric mice inoculated with DAA-naïve hepatitis C patient sera carrying L31-Y93 wild type HCV rapidly declined to undetectable levels following 4 weeks of LDV/GS-558093 treatment^18^. Afterwards, the HCV RNA levels of these mice were followed long-term, and 2 of 4 mice experienced relapse after the end of treatment. Deep sequencing was performed at week 10 (6 weeks after the end of LDV/GS-558093 treatment) in one mouse with relapse (Fig. 2A); the L31-Y93H single substitution was detected as a major clone (90.1%) and the L31V-Y93 single substitution or L31V-Y93H double substitution were detected as minor clones (4.0% or 0.9%, respectively) (Table 2). Four weeks later (at week 14), deep sequencing was performed again and revealed that the L31-Y93H single substitution was increased to 99.4%, while both the L31V-Y93 single substitution and the L31V-Y93H double substitution were decreased to undetectable levels. From week 14, the mouse was re-treated with ASV/DCV for 4 weeks, and the HCV RNA levels decreased to undetectable levels but relapsed again at 4 weeks after the end of re-treatment (Fig. 2A). At week 26 (8 weeks after the end of ASV/DCV re-treatment), deep sequencing was performed and revealed that the L31V-Y93H double substitution had emerged with a high frequency of 98.6% and that it had been maintained as a major clone long-term until week 31. To investigate the origin of L31V-Y93H double substitution after ASV/DCV re-treatment, a phylogenetic tree was constructed using the sequencing data at week 10 (LDV/GS-558093 after 6 weeks, named group A), week 14 (LDV/GS-558093 after 10 weeks, named group B), and week 26 (ASV/DCV after 8 weeks, named group C). The top 10 clusters in each group and the top 1 cluster from the minor L31V-Y93H double substitution in group A (Am1) were analysed in the phylogenetic tree. The L31-Y93H single substituted clusters in group B, such as B3, B4 and B5, were closely related to the L31V-Y93H double substituted clusters in group C; however, the cluster in Am1 was relatively far from the clusters in group C (Fig. 2B). This result suggested that the major clone of the L31V-Y93H double substitution after ASV/DCV re-treatment was derived from the major clone of the L31-Y93H single substitution after LDV/GS-558093 with the additional L31V substitution but not from the minor clone of the L31V-Y93H double substitution, which was present at the early phase following LDV/GS-558093 but was later reduced to undetectable levels.

### NS5A RAS generation during DAA treatment in full-genome HCV RNA-injected mice

**Figure 3:**
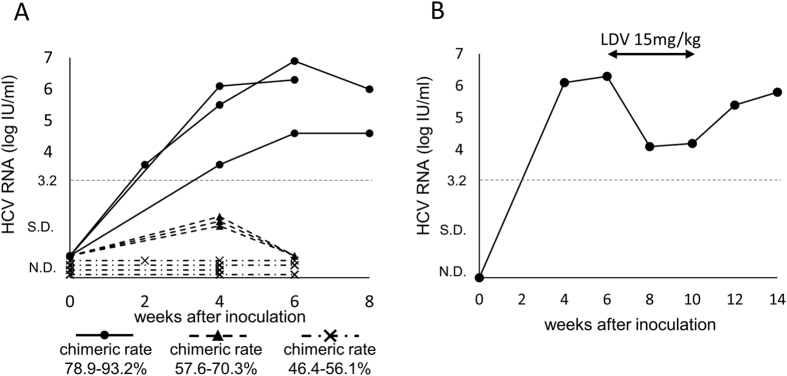
Generating new NS5A mutation in mouse. (**A**) Serum HCV RNA levels in human hepatocyte chimeric mice inoculated with engineered full-genome HCV RNA from the wild type Ly strain via intrahepatic injection. Closed circle with a solid line, closed triangle with a dashed line and cross-mark with a dashed line represent the high chimeric rate mice (chimeric rate 78.9–93.2%), moderate chimeric rate mice (chimeric rate 57.6–70.3%) and relatively low chimeric rate mice (chimeric rate 46.4–56.1%), respectively. (**B**) Serum HCV RNA levels in a human hepatocyte chimeric mouse that was persistently infected with full-genome HCV RNA. The mouse was treated with 4 weeks of LDV monotherapy and deep sequencing was performed before and after treatment (at weeks 6 and 14). N.D., not detected, and S.D., signal detected but at lower quantification levels.

To confirm the possibility of adding a new mutation during DAA treatment in an *in vivo* environment without quasispecies, engineered monoclonal full-genome HCV RNA (HCV-Ly strain, genotype 1b; both NS5A L31 and Y93 are completely wild type) was intrahepatically injected into human hepatocyte chimeric TK-NOG mice in 400 μl of phosphate-buffered saline (PBS). The HCV RNA levels in mice sera from chimeric mice with a high human hepatocyte chimeric rate (78.9% to 98.2%) gradually increased, and a persistent infection was established (Fig. 3A and Table 3). The HCV RNA in sera from chimeric mice with a moderate chimeric rate (57.6% to 72.8%) was transiently detected but decreased to undetectable levels afterwards. Finally, the HCV RNA was never detected in sera from chimeric mice with a relatively low chimeric rate (46.4% to 56.1%). Thus, we established continuous HCV infection in highly chimeric mice via intrahepatic injection with the monoclonal full-genome HCV RNA. Six weeks after injecting the full-genome RNA into chimeric mice, one of the persistently infected mice was analysed by deep sequencing. The wild type L31-Y93 clones represented 99.5% of the HCV clones, and L31V/M or Y93H substitutions were not detected (Table 4, Supplementary Table 2). The mouse was then treated with LDV monotherapy for 4 weeks. The HCV-RNA levels in the mouse serum were reduced by 2 log IU/ml but did not achieve an undetectable level. They increased again after the end of LDV treatment (Fig. 3B). Four weeks after the end of LDV treatment, deep sequencing was performed again and revealed that the L31-Y93H single substitution was newly generated and represented 99.5% of the HCV clones (Table 4, Supplementary Table 2).

## Discussion

In this study, we investigated the origin of a L31M/V-Y93H double substitution after VF with DAA treatment that included NS5A inhibitors. We used deep sequencing without fragmentation and phylogenetic tree analysis combined with clustering by CD-HIT. Deep sequencing without fragmentation enabled us to sequentially read the base alignments up to 400 bases using Ion PGM^19^, which contributed to a novel investigation to calculate the frequency of the L31M/V-Y93 single substitution, L31-Y93H single substitution and L31M/V-Y93H double substitution. Phylogenetic tree analysis is useful for estimating the genetic distance between various HCV strains and is represented by the total length of the lines that connect each strain in the phylogenetic tree^20^,^21^. Using CD-HIT to cluster the HCV clones that display the same base alignments, we could analyse the most frequent or important clones and improve the accuracy and quantity of information in the constructed phylogenetic trees.

In the investigation with the ASV/DCV non-SVR patients, the baseline L31M/V-Y93H double substitution in cases 1 and 2 was present at over 10% frequency, and the phylogenetic tree analysis suggested that this double substituted variant was the origin of the double substitution after VF. This result was understandable considering the high resistance of L31M/V-Y93H double substituted variants. Additionally, in cases 3 and 4, the double substituted variants at baseline were also considered to be the putative origin of the double substitution after VF despite a lower than 1% frequency of the double substitution at baseline. These results suggested that pre-existing minor L31M/V-Y93H double substituted variants could occasionally contribute to the double substitution after VF despite a frequency of less than 1%.

In cases 5 to 8, the L31-Y93H single substituted variants or L31-Y93 wild clones were considered to be the origin of the double substitution after VF. These results suggested that new L31M/V or L31M/V plus Y93H substitutions could be newly generated during DAA treatment in L31-Y93H single substituted variants or L31-Y93 wild clones. Notably, the L31-Y93H single substituted variants in case 7 were considered to be the origin of the double substitution after VF despite a L31-Y93H frequency as low as 3.0%. Thus, not only the minor clone for the L31M/V-Y93H double substitution but also the minor clone for the L31-Y93H single substitution could contribute to the emergence of the double substitution after VF. In cases 9 and 10, the phylogenetic tree was not constructed because there was no single or double substitution at baseline; however, we speculated that new L31M plus Y93H substitutions were added to pre-existing wild clones, leading to the emergence of the L31M-Y93H double substitution after VF. However, due to the limited detection sensitivity of deep sequencing, the possibility remains; extremely rare double substituted variants may exist at undetectable levels at baseline and increase during DAA treatment in such cases.

We assessed such a possibility in the following experiment using chimeric mice treated with LDV/GS-558093 and ASV/DCV. The phylogenetic tree analysis of the chimeric mice suggested that the major L31-Y93H single substituted variants rather than transient minor L31V-Y93H double substituted variants after the first therapy were the putative origin of the double substitution after ASV/DCV re-treatment. At the time of the ASV/DCV re-treatment, the transient L31V-Y93H double substituted variants were reduced to undetectable levels, however, these variants may exist at levels lower than the detection limit. Taken together, the L31V-Y93H double substituted variants below the detection limit of deep sequencing may not significantly impact the emerging RAS after DAA treatment.

In the final section, we established monoclonal HCV-infected mice via intrahepatic injection of full-genome HCV RNA. This *in vivo* model is a useful platform to investigate the characteristics of HCV without quasispecies. Using these mice, we clearly demonstrated that it is possible to generate the Y93H mutation in completely wild clones via DAA treatment in an *in vivo* situation despite no quasispecies presence. This result supports the speculation regarding clinical cases 5 to 10 that new substitutions of L31M and/or Y93H were added to pre-existing wild clones. This mechanism of adding new mutations during DAA treatment could be caused by the high mutation rate in replicating HCV RNA. Mice with a high chimeric rate (greater than 80%) were required to develop a persistent HCV infection via the intrahepatic injection of full-genome HCV RNA, while chimeric mice with a chimeric rate over 40% developed sustained HCV infection by inoculation with HCV patient sera^18^. Thus, there are higher hurdles to establish a sustained infection with full-genome HCV RNAs. This *in vivo* model could also be applicable in examining the pure influence of a specific mutation on HCV by introducing site-directed mutations into the full-genome RNA.

In the present study, 7 of 11 non-SVR patients with ASV/DCV had L31M/V-Y93H double substitutions at baseline. However, it remained unclear whether pre-existing minor clones for L31M/V-Y93H double substitutions have an impact on the SVR rate of DAA treatment that includes an NS5A inhibitor. The major clones for baseline L31M/V or Y93H by direct sequencing decreased the SVR rate in ASV/DCV treatment^2^,^3^,^22^; however, minor clones of these substitutions did not affect the therapeutic efficacy^23^. Additionally, excluding HCV patients with minor substitutions in NS5A (less than 20%) using deep sequencing did not improve the SVR rate in ASV/DCV therapy^24^. However, the existence of the L31M/V-Y93H double substitution was not analysed. Interestingly, in as many as 4 patients in this study, the L31M/V-Y93H double substituted variants at baseline were suggested to contribute to the double substitution after VF. To assess the impact of pre-existing L31M/V-Y93H minor clones on the SVR rate in DAA treatment, further studies comparing the frequency of baseline L31M/V-Y93H double substitutions between SVR and non-SVR patients are needed.

In conclusion, both the selection of pre-existing substituted variants in quasispecies and the generation of new mutations during DAA treatment are important mechanisms for emerging RASs of HCV in non-SVR patients. Pre-existing minor substitutions, such as L31-Y93H or L31M/V-Y93H, occasionally influence emerging double substitutions after VF.

## Materials and Methods

### Patients and dual oral therapy with ASV/DCV

Of the 322 hepatitis C patients who received dual oral therapy of ASV (Sunvepra; Bristol-Myers Squibb, New York, NY, at 200 mg/day twice per day) plus DCV (Daklinza; Bristol-Myers Squibb, at 60 mg/day once per day) for 24 weeks from September 2014 to August 2015 at Osaka University Hospital and other institutions participating in the Osaka Liver Forum (OLF), 14 patients with genotype 1b who had never undergone DAA treatment developed VF with ASV/DCV treatment. We analysed the 14 patients in this study using deep sequencing followed by phylogenetic tree analysis. This study was conducted according to the ethical guidelines of the Declaration of Helsinki amended and was approved by the ethics commission of Osaka University Hospital and the institutional review boards of the participating hospitals. Written informed consent was obtained from all patients.

### HCV infection in human hepatocyte chimeric mice

NOG mice expressing a thymidine kinase transgene (TK-NOG)-based human hepatocyte chimeric mice were generated as previously reported^25^. Chimeric mice with a human hepatocyte chimeric rate of greater than 40%, which was calculated based on the human albumin concentration in the mice serum, were used in this experiment. Human serum albumin levels were measured using ELISA with the Human Albumin ELISA Quantitation Set (Bethyl Laboratories Inc., TX). The mice were infected with HCV using the following two methods: (1) intravenous injection via tail vein with 100 μl of hepatitis C from a patient serum (DAA-naïve, genotype 1b, 6.8 log IU/ml) or (2) intrahepatic injection with 100 μg of engineered full-genome HCV RNA (Ly-HCV^26^, wild type from genotype 1b HCV, accession No. AB779562) in 400 μl of PBS. After inoculation, the mice sera were collected every 1 to 4 weeks and the HCV RNA levels were measured with the COBAS TaqMan HCV test (Roche Diagnostics, Basel, Switzerland) in 100-fold diluted serum (with a lower measurement range of 3.2 log IU/ml serum). The hepatitis C patient serum, which was used as an inoculum for the chimeric mice, was collected after obtaining written informed consent under the approval of the Osaka University Hospital Ethics Committee.

### Engineering full-genome HCV RNA (Ly-HCV)

To generate the full-length HCV construct, patient serum from which Ly-HCV could be isolated was used. Total RNA was extracted from 140 μL of patient serum by using the QIAamp Viral RNA kit (QIAGEN, Valencia, CA), and cDNA was synthesized using Superscript IV (Invitrogen, Carlsbad, CA) with a random 6-mer primer. Nearly the entire open reading frame as well as a portion of the 5′-untranslated region (UTR) of the synthesized cDNA was subsequently amplified by using nested PCR with TaKaRa LA Taq DNA polymerase (Takara Bio, Shiga, Japan). Appropriate PCR primers were used to amplify 9 fragments of the HCV cDNA (nt. 120–1324, nt. 1002–2000, nt. 1879–2634, nt. 2556–3680, nt. 3531–4645, nt. 4468–5320, nt. 5140–7168, nt. 7006–8100, and nt. 7821–9380). The sections of the 5′-UTR and 3′-UTR not accounted for in the above PCR reactions were synthesized by referring to the consensus sequence of the HCV genotype 1b strain. By using the amplified and generated fragments as templates, 6 primary fragments for the full-length Ly-HCV were produced (nt. 1–178, nt. 178–1990, nt. 1990–3638, nt. 3638–5305, nt. 5305–6465, nt. 6465–7823, and nt. 7823–9644). The plasmid containing the full-length Ly-HCV was constructed by digesting the fragments with restriction enzymes as follows: *Mfe*I at the 5′-end, *Age*I at nt. 178, *Not*I at nt. 1990, *Sfi*I at nt. 3638, *Nsi*I at nt. 5305, *Bgl*II at nt. 6465, *Afl*II at nt. 7823, and *Xba*I at the 3′-end. The sequence of the constructed plasmid was confirmed and corrected to maintain the amino acid sequence of Ly-HCV by site-directed PCR when PCR-associated mutations manifested. The full-length HCV RNAs of these strains were synthesized as previously described^27^.

### Treatment of HCV-infected mice with DAAs

After a stable HCV infection was established in the mice, they were administered DAA therapy orally once per day as follows. Mice inoculated with HCV from patient serum were treated with a dual therapy of ledipasvir (LDV, 15 mg/kg)/GS-558093 (50 mg/kg, nucleotide analogue NS5B polymerase inhibitor) (LDV and GS-558093 were kindly provided by Gilead Sciences Inc., Foster City, CA). A mouse that experienced relapse after LDV/GS-558093 was re-treated with asunaprevir (40 mg/kg)/daclatasvir (30 mg/kg) (ASV and DCV were kindly provided by Bristol-Myers Squibb) for 4 weeks. A mouse inoculated with the full genome HCV RNA was treated with a monotherapy of LDV (15 mg/kg) for 4 weeks. All experimental protocols were approved by the Animal Care and Use Committee of Osaka University Medical School and the Animal Care Committee of Central Institute for Experimental Animals (CIEA), and all experiments were performed according to approved protocols.

### Deep sequencing analysis

Deep sequencing analysis was performed in the same manner as we previously reported^18^. Briefly, HCV RNA was extracted from human or mouse serum samples using QIAamp Viral RNA Mini (QIAGEN, Hilden, Germany) and reverse transcribed with HCV-specific whole reverse primers using Super ScriptTMIII Reverse Transcriptase (Thermo Fisher Scientific Inc., MA). The NS5A region was amplified with outer primer pairs using KOD-Plus-Neo (Toyobo, Osaka Japan). The first amplicons were further amplified using Platinum PCR SuperMix High Fidelity (Thermo Fisher Scientific Inc.) with inner primer pairs, which were attached with the adaptors and barcodes. After purification with AMPure (Beckman Coulter, CA), the second amplicons were sequenced without fragmentation with the Ion Personal Genome Machine (Ion PGM) Sequencer (Thermo Fisher Scientific Inc.). The NS5A L31 and Y93 positions were analysed continuously in one amplicon. The coverage of each sequence was at least more than 5000 reads. To validate the deep sequencing error rates, we performed a control experiment for three runs using plasmids encoding wild type HCV. Cut-off values were calculated as the mean error rates +2SD and determined as 0.20%, 0.22% and 0.10% for the L31M/V-Y93 single substitution, L31-Y93H single substitution and L31M/V-Y93H double substitution, respectively (Supplementary Table 1).

### Phylogenetic tree analysis

The leads from each of the samples analysed by deep sequencing were aligned, and leads with the same base alignment were gathered into one cluster using CD-HIT^28^ (with a 100% concordance threshold). The number of reads in each cluster was counted and sorted in ascending order, and the top 10 most frequent clusters for each sample were extracted. In cases with baseline minor clones of L31M/V-Y93, L31-Y93H and L31M/V-Y93H substituted variants that were not included within the top 10 clusters, the minor substituted variants were extracted separately and clustered using CD-HIT in the same manner. The phylogenetic trees were constructed with the top 10 clusters from each sample and the top minor mutant clusters in some cases, using the neighbour-joining method with MEGA 7^29^,^30^. Bootstrapping was performed with 1000 replicates to confirm reliability.

## Additional Information

**How to cite this article**: Kai, Y. *et al*. Baseline quasispecies selection and novel mutations contribute to emerging resistance-associated substitutions in hepatitis C virus after direct-acting antiviral treatment. *Sci. Rep.* **7**, 41660; doi: 10.1038/srep41660 (2017).

**Publisher's note:** Springer Nature remains neutral with regard to jurisdictional claims in published maps and institutional affiliations.

## Supplementary Material

Supplementary Figure and Tables

## Figures and Tables

**Table 1:** NS5A L31/Y93 RASs at baseline and after failure in patients treated with ASV/DCV.

Case	Baseline (A)				Result	After failure (B)			
	L31-Y93	L31M/V-Y93	L31-Y93H	L31M/V-Y93H		L31-Y93	L31M/V-Y93	L31-Y93H	L31M/V-Y93H
1	2.3%	M 57.0%	H 0.8%	M-H 38.9%*	relapse	—	—	—	M-H 99.0%
2	25.9%	V 3.2%	H 57.6%	V-H 12.9%*	relapse	—	—	—	V-H 98.2%
3	97.6%	M 0.4%	H 0.3%	M-H 0.1%*	breakthrough	—	—	H 0.7%	M-H 97.5%
4	—	—	H 99.2%	V-H 0.1%*	relapse	—	—	—	V-H 99.4%
5	—	—	H 96.2%*	V-H 0.3%	breakthrough	—	—	—	V-H 97.7%
6	39.1%	—	H 59.8%*	M-H 0.3%	relapse	1.5%	—	H 0.7%	M-H 97.0%
7	94.6%	—	H 3.0%*	—	breakthrough	—	—	—	V-H 93.0%
8	98.6%*	M 0.7%	—	—	relapse	—	—	—	V-H 53.7%
									M-H 43.9%
9	98.8%	—	—	—	relapse	—	—	—	M-H 93.8%
10	97.9%	—	—	—	relapse	—	—	H 0.6%	M-H 98.2%
11	99.0%	—	—	M-H 0.2%	breakthrough	99.5%	—	—	—

^*^Represents the putative original variants before treatment that contributed to the L31M/V-Y93H double substitution after treatment.

**Table 2:** NS5A L31/Y93 RASs in mouse administrated with LDV/GS-558093 treatment and ASV/DCV re-treatment.

	L31-Y93	L31V-Y93	L31-Y93H	L31V-Y93H
week 0	95.9%	—	—	—
week 10 (A)	3.0%	4.0%	90.1%	0.9%
(LDV/GS-558093 post 6 w)				
week 14 (B)	—	—	99.4%*	—
(LDV/GS-558093 post 10 w)				
week 26 (C)	—	—	0.7%	98.6%
(ASV/DCV post 8 w)				
week 29	—	—	17.4%	75.4%
(ASV/DCV post 11 w)				
week 31	—	—	9.0%	82.9%
(ASV/DCV post 13 w)				

^*^Represents the putative original variants before treatment that contributed to the L31M/V-Y93H double substitution after treatment.

**Table 3:** Relationship between human hepatocyte chimeric rates of mice and HCV RNA detection in mice sera after intrahepatic injection of full-genome HCV RNAs.

Chimeric rates of mice	HCV RNA detection
93.2%	persistently detected
81.5%	persistently detected
78.9%	persistently detected
70.3%	transiently detected
58.7%	transiently detected
57.6%	transiently detected
56.1%	never detected
54.3%	never detected
51.1%	never detected
46.4%	never detected

**Table 4:** NS5A L31/Y93 RASs in mouse inoculated with full-genome HCV RNA followed by 4 weeks of LDV monotherapy.

	L31-Y93	L31V-Y93	L31-Y93H	L31V-Y93H
week 6	99.5%	—	—	—
(before treatment)				
week 14	0.3%	—	99.5%	—
(LDV post 4 w)				
