# Supplementary material for: Baseline quasispecies selection and novel mutations contribute to emerging resistance-associated substitutions in hepatitis C virus after direct-acting antiviral treatment
Source: Sci Rep. 2017 Jan 30;7:41660. doi: 10.1038/srep41660 (PMC5278351; doi:10.1038/srep41660)
Supplement: Supplementary Figure and Tables [file srep41660-s1.pdf]

Baseline quasispecies selection and novel mutations contribute to emerging resistance-associated substitutions in hepatitis C virus after direct-acting antiviral treatment

Yugo Kai, Hayato Hikita, Naoki Morishita, Kazuhiro Murai, Tasuku Nakabori, Sadaharu Iio, Hideki Hagiwara, Yasuharu Imai, Shinji Tamura, Syusaku Tsutsui, Masafumi Naito, Meiko Nishiuchi, Yasuteru Kondo, Takanobu Kato, Hiroshi Suemizu, Ryoko Yamada, Tsugiko Oze, Takayuki Yakushijin, Naoki Hiramatsu, Ryotaro Sakamori, Tomohide Tatsumi, Tetsuo Takehara.

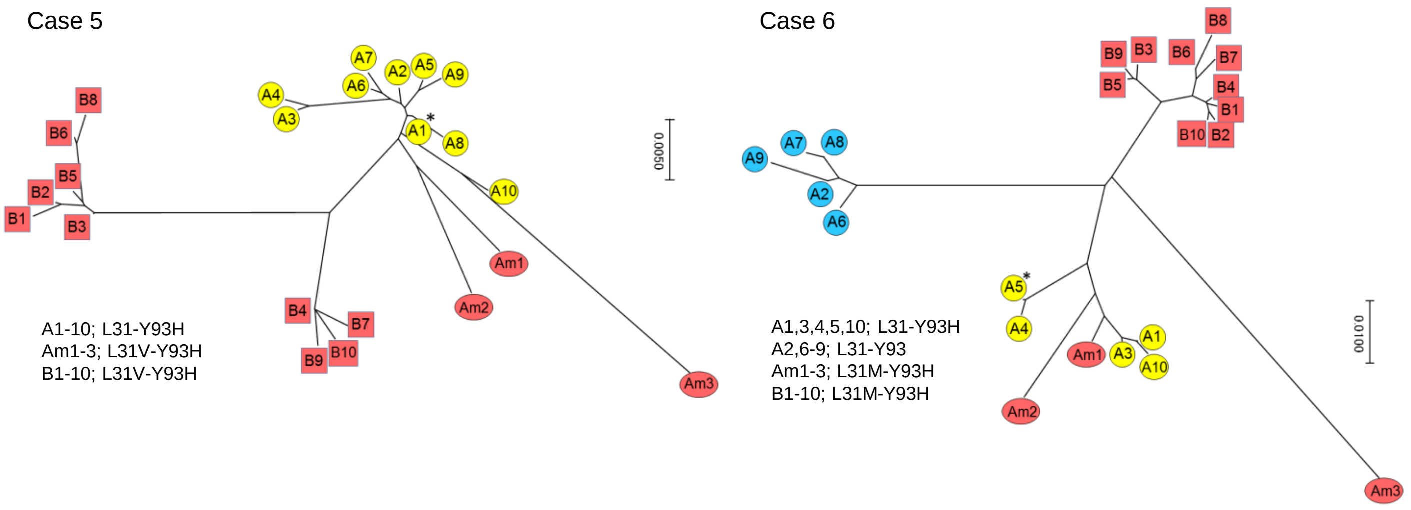

Supplementary Figure 1.
Phylogenetic tree analysis including further minor clusters of pre-existing L31M/V-Y93H variant in case 5 and case 6. In both case, pre-existing minor L31M/V-Y93H substituted clusters consisted of more than 3 leads (Am1, Am2 and Am3) were added to phylogenetic tree. The top 10 clusters from the HCV clones at baseline are represented as A1 to A10 with coloured circles, and the top 10 clusters at VF are represented as B1 to B10 with coloured squares. Blue, yellow and red colours represent the L31-Y93 wild type, L31M/V or Y93H single substitution and L31M/V-Y93H double substitution, respectively. *; represents the putative original cluster before treatment that contributed to the L31M/V-Y93H double substitution after treatment.

Supplementary Table 1. The control experiment to determine the error rates of deep sequencing using plasmid encoding a wild type HCV.

Variant	Sample	Error rate (%)	Error rate Mean ± SD (%)	Cut-off value Mean + 2SD (%)
L31-Y93H	1	0.16	0.16 ± 0.03	0.22
	2	0.19		
	3	0.13		
L31M/V-Y93	1	0.05	0.06 ± 0.07	0.20
	2	0.13		
	3	0.00		
L31M/V-Y93H	1	0.06	0.04 ± 0.03	0.10
	2	0.06		
	3	0.00		
L31-Y93S	1	0.04	0.01 ± 0.02	0.05
	2	0.00		
	3	0.00		

Supplementary Table 2. NS5A variants focusing on amino acid at L31 and Y93 in mouse inoculated with full-genome HCV RNA followed by 4 weeks of LDV monotherapy.

	Variants	Frequency
week 6 (before treatment)	L31-Y93	99.5%
	L31-Y93S	0.3%
	other variants	under cut-off value
week 14 (LDV post 4 weeks)	L31-Y93H	99.5%
	L31-Y93	0.3%
	other variants	under cut-off value
